# Correction: Ou et al. Characteristic Microbiomes Correlate with Polyphosphate Accumulation of Marine Sponges in South China Sea Areas. *Microorganisms* 2020, *8*, 63

**DOI:** 10.3390/microorganisms9091826

**Published:** 2021-08-27

**Authors:** Huilong Ou, Mingyu Li, Shufei Wu, Linli Jia, Russell T. Hill, Jing Zhao

**Affiliations:** 1 College of Ocean and Earth Science of Xiamen University, Xiamen 361005, China; hlou@xmu.edu.cn (H.O.); mingyuli@stu.xmu.edu.cn (M.L.); 22320151152141@stu.xmu.edu.cn (S.W.); 22320162201046@stu.xmu.edu.cn (L.J.); 2 Institute of Marine and Environmental Technology, University of Maryland Center for Environmental Science, Baltimore, MD 21202, USA; 3 Xiamen City Key Laboratory of Urban Sea Ecological Conservation and Restoration (USER), Xiamen University, Xiamen 361005, China

The authors wish to make the following corrections to this paper [1]:

There is a mistake in a unit of DIP in Surrounding Sea Water provided in Table 1. The unit of DIP in Surrounding Sea Water listed in the original version of the article was “mM”.

The correct version should be as follows:

The correct unit of DIP in Surrounding Sea Water in Table 1 is “µM”.

The authors would like to apologize for any inconvenience caused to the readers by these changes.
