# Supplementary material for: Characteristic Microbiomes Correlate with Polyphosphate Accumulation of Marine Sponges in South China Sea Areas
Source: Microorganisms. 2019 Dec 30;8(1):63. doi: 10.3390/microorganisms8010063 (PMC7022310; doi:10.3390/microorganisms8010063)
Supplement: Supplementary file 1 [file microorganisms-08-00063-s001.zip › Supplementary Materials/supplementary materials.docx]

**Table S1.** ppk1 gene primers and the reaction conditions for PCR*

| Primer | Sequence (5’-3’) | Reaction conditions | length |
| --- | --- | --- | --- |
| ppk1 | F: GGSGGYCGKTAYCAYAAYTTYAARGACTTYATT  R: ATATTGCGCGTCATCCAGTCSGC | 94 °C for 5 min; 35 cycles of 94 °C for 1 min, 57 °C for 45 s, and 72 °C for 2 min; and a final extension at 72 °C for 12 min | 1000bp |
| ppk2 | F: GARTTKMGHGCRMGCTTCGAYGA  R: GTGYTCCARAAAGCKSCC | 94 °C for 5 min; 35 cycles  of 94 °C for 1 min, 56 °C for 45 s, and 72 °C for 2 min; and a final extension at 72 °C for 12 min | 600bp |

*Primers were designed with Primer Premier 5.

**Table S2.** 16S rRNA gene amplicon sequence summary and estimation of richness and diversity for sponge samples

| Sample  Name | Observed species | Richness estimators | | Diversity indices | | Observed_species/expected_species |
| --- | --- | --- | --- | --- | --- | --- |
|  |  | Chao1 | ACE | Shannon | Simpson |  |
| DM-1 | 410 | 737.842 | 1070.706 | 1.559 | 0.540 | 0.383 |
| DM-2 | 457 | 715.714 | 901.705 | 2.418 | 0.223 | 0.507 |
| DM-3 | 314 | 745.068 | 1001.958 | 2.178 | 0.311 | 0.313 |
| DC-1 | 446 | 568.667 | 610.753 | 4.063 | 0.871 | 0.779 |
| DC-2 | 447 | 548.369 | 560.268 | 3.952 | 0.87 | 0.778 |
| DC-3 | 543 | 647.227 | 701.31 | 4.229 | 0.863 | 0.774 |
| DT-1 | 582 | 666.867 | 677.159 | 4.409 | 0.881 | 0.860 |
| DT-2 | 493 | 576.135 | 605.333 | 4.179 | 0.885 | 0.814 |
| DT-3 | 536 | 687.11 | 737.601 | 4.197 | 0.875 | 0.727 |
| CH01-1 | 559 | 872.544 | 854.122 | 4.183 | 0.861 | 0.654 |
| CH01-2 | 556 | 816.682 | 803.192 | 4.104 | 0.856 | 0.692 |
| CH01-3 | 561 | 900.082 | 898.020 | 4.144 | 0.860 | 0.625 |
| CH02-1 | 949 | 1218.444 | 1204.9 | 5.425 | 0.890 | 0.788 |
| CH02-2 | 917 | 1372.327 | 1307.979 | 4.914 | 0.848 | 0.701 |
| CH02-3 | 937 | 1378.052 | 1340.093 | 5.067 | 0.854 | 0.700 |
| LQ01-1 | 1018 | 1570.025 | 1522.437 | 5.124 | 0.874 | 0.669 |
| LQ01-2 | 1020 | 1620.624 | 1573.766 | 4.979 | 0.859 | 0.648 |
| LQ01-3 | 881 | 1285 | 1270.224 | 4.762 | 0.851 | 0.694 |
| LQ02-1 | 707 | 1078.172 | 965.237 | 5.809 | 0.917 | 0.732 |
| LQ02-2 | 656 | 948.125 | 1010.912 | 4.961 | 0.886 | 0.649 |
| LQ02-3 | 722 | 969.04 | 944.869 | 5.692 | 0.911 | 0.764 |
| MX01-1 | 889 | 1130.875 | 1084.144 | 6.131 | 0.931 | 0.820 |
| MX01-2 | 1308 | 1562.407 | 1539.196 | 6.402 | 0.935 | 0.850 |
| MX01-3 | 1241 | 1584.492 | 1563.29 | 5.540 | 0.893 | 0.794 |
| MX02-1 | 575 | 1109.444 | 1075.083 | 5.429 | 0.958 | 0.535 |
| MX02-2 | 480 | 779.845 | 788.867 | 5.332 | 0.955 | 0.608 |
| MX02-3 | 511 | 852.714 | 860.588 | 5.300 | 0.954 | 0.594 |
| MX03-1 | 421 | 658.109 | 649.941 | 3.889 | 0.847 | 0.648 |
| MX03-2 | 611 | 1067.278 | 1041.169 | 4.090 | 0.858 | 0.587 |
| MX03-3 | 553 | 1016.219 | 959.229 | 4.120 | 0.852 | 0.577 |
| QZ01-1 | 435 | 718.2 | 692.598 | 2.943 | 0.746 | 0.628 |
| QZ01-2 | 417 | 607.318 | 655.560 | 3.115 | 0.767 | 0.636 |
| QZ01-3 | 565 | 726.717 | 738.919 | 3.412 | 0.812 | 0.765 |
| QZ02-1 | 497 | 695.154 | 684.146 | 3.519 | 0.747 | 0.726 |
| QZ02-2 | 359 | 584.213 | 617.306 | 2.808 | 0.665 | 0.582 |
| QZ02-3 | 546 | 714.765 | 713.037 | 3.569 | 0.742 | 0.766 |

**Table S3.** Test of alpha-diversity indices in different samples

| **Index** | **Groups** | **Test method** | | **P-value** | **Significance** |
| --- | --- | --- | --- | --- | --- |
| Chao1 | CH01-VS-CH02 | | T-test | 0.0050 | ** |
| Chao1 | LQ01-VS-LQ02 | | Tukey HSD | 0.0043 | ** |
| observed_species | MX01-VS-MX02-VS-MX03 | | Tukey HSD | 0.0044 | ** |
| Shannon | LQ02-VS-MX01 | | T-test | 0.0373 | * |
| Shannon | MX02-VS-QZ02 | | T-test | 0.0124 | * |

**
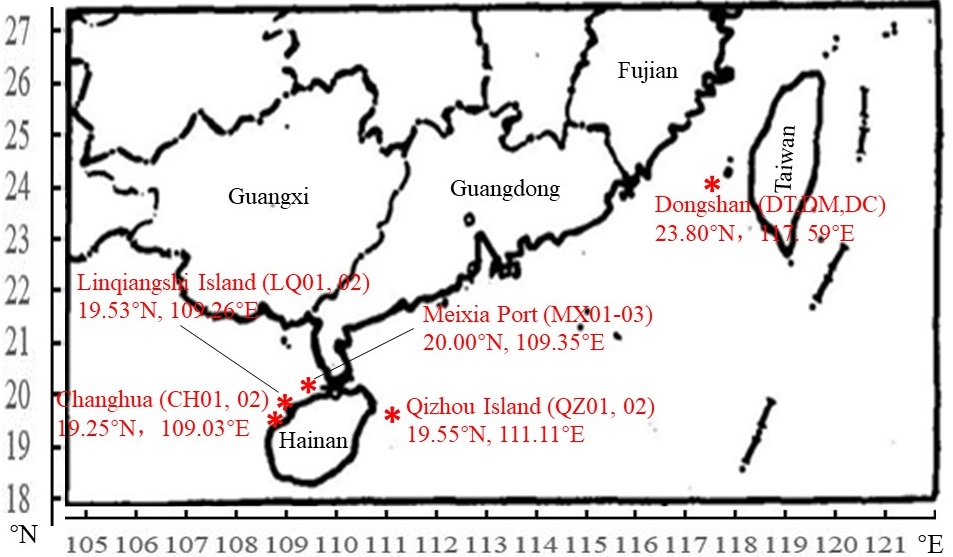
Figure S1.** Sampling sites of sponges. (Sponges ID in the brackets)


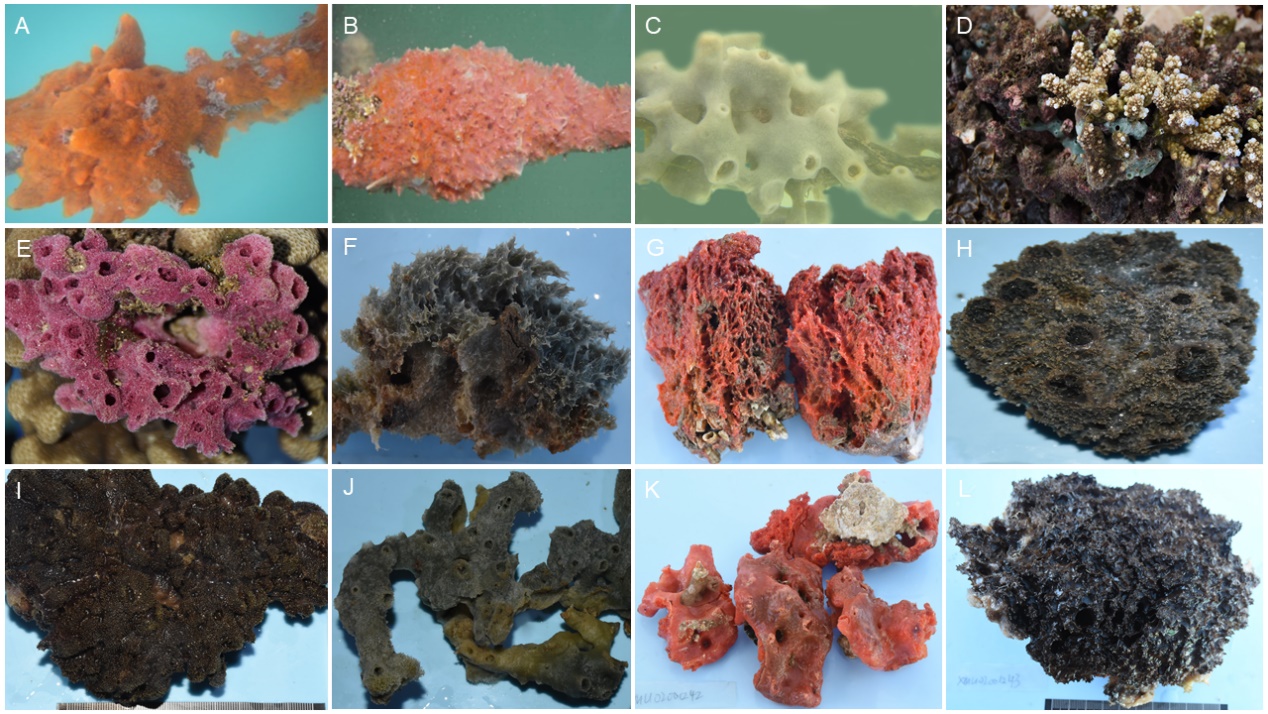


**Figure S2.** Pictures of 12 sponge species. A. DT (*Tedania* sp.); B. DM (*Mycale* sp.); C. DC (*Callyspongia* sp;); D. CH01 (*Haliclona* sp.); E. CH02 (*Cladocroce* sp.); F. LQ01 (*Lissodendoryx* sp.); G. LQ02 (*Mycale* sp.); H. MX01 (*Lissodendoryx* sp.); I. MX02 (*Ircinia* *dendroides*); J. MX03 (*Callyspongia* sp.); K. QZ01 (*Sigmaxinella* sp.); L. QZ02 (*Ircinia* sp.).

**
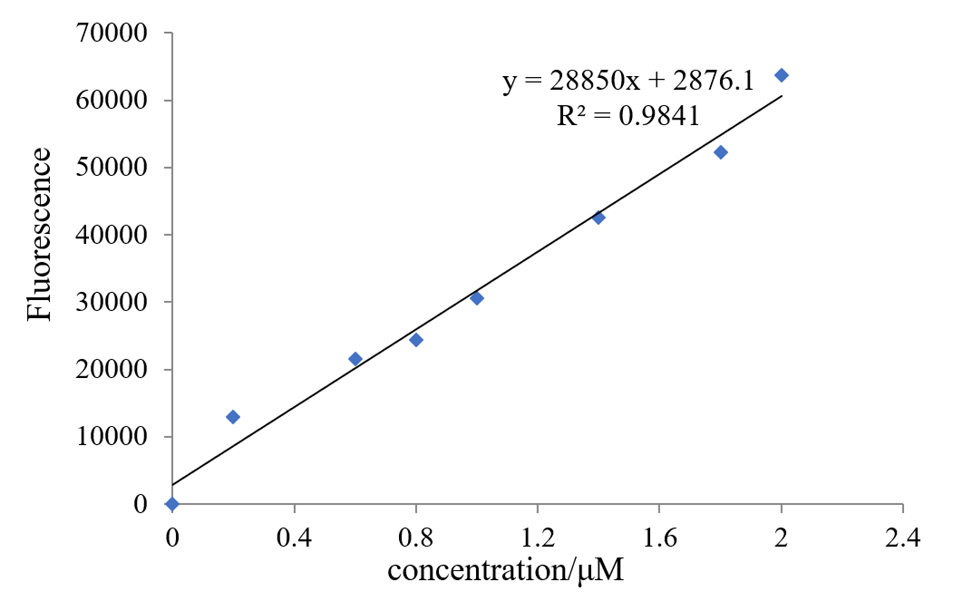
****Figure S3.** polyP standard curve

**
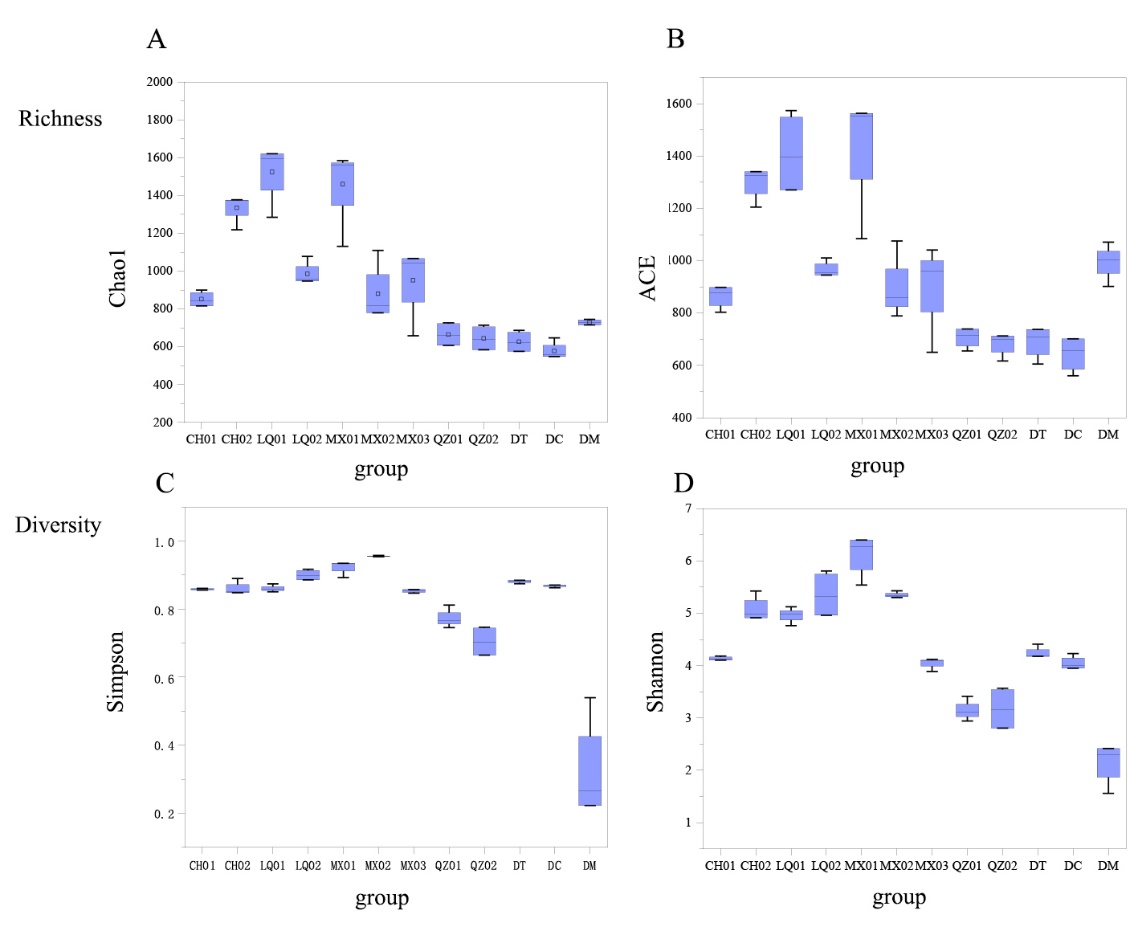
Figure S4.** Microbial community diversity and richness associated with 12 sponges. A. Box plot of chao1 indexes; B. Box plot of ACE indexes; C. Box plot of simpson indexes; D. Box plot of shannon indexes.

**
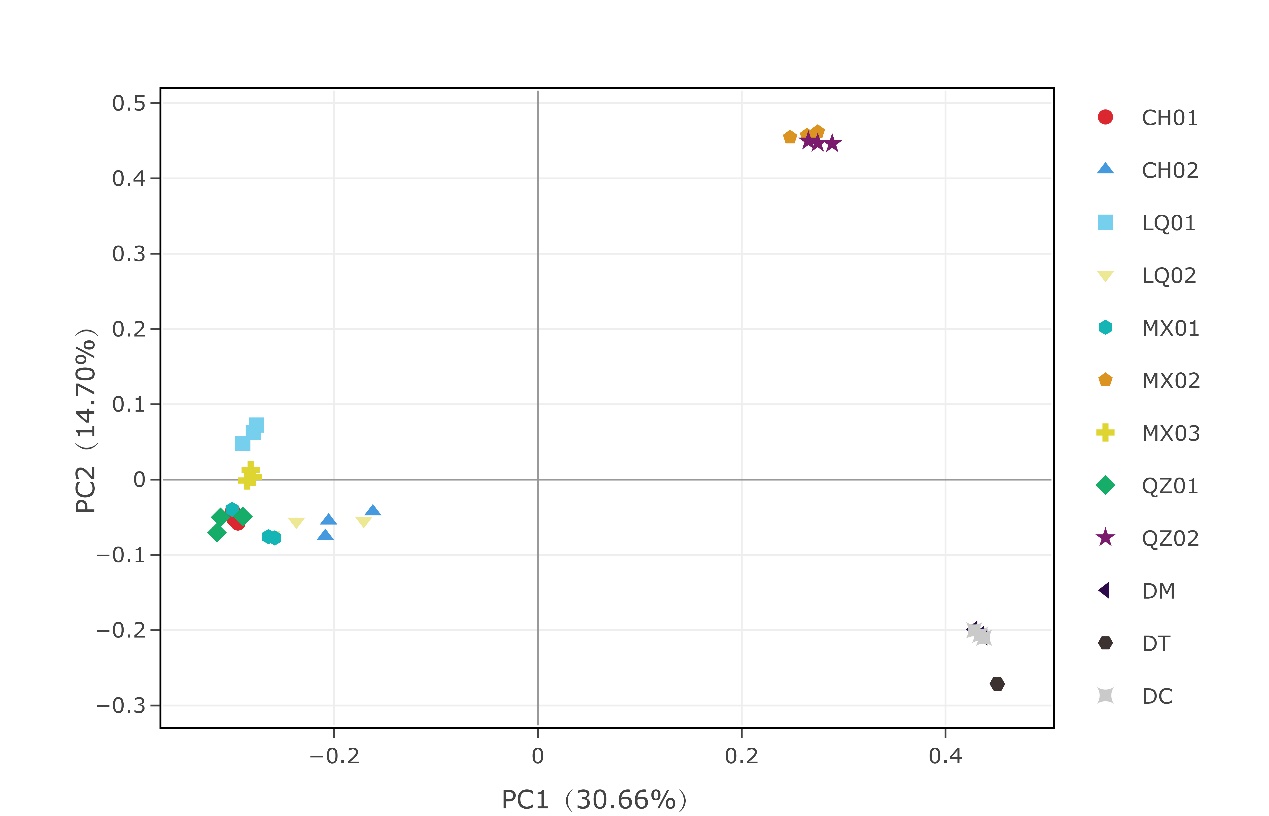
Figure S5.** The principle coordinates analysis (PCoA) of microbiome associated with 12 sponge samples.

**
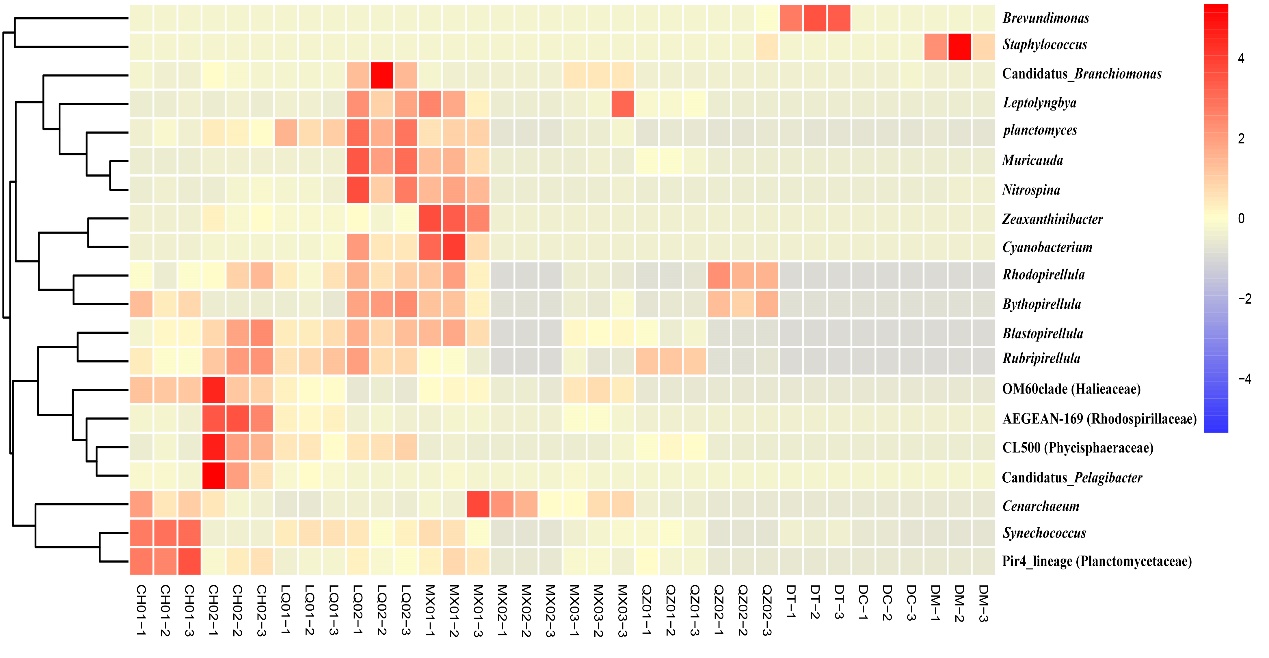
**

**Figure S6.** Heatmap of the distribution of bacterial representatives (richness top 20) among different stages at the level of order. The color code indicates relative abundance, ranging from blue (low abundance) to yellow to red (high abundance).

**
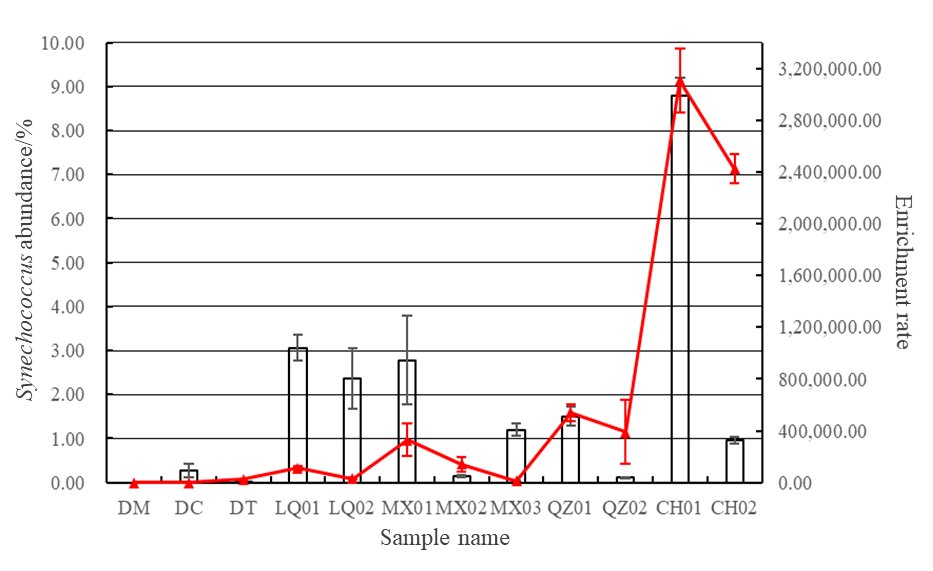
Figure S7.** The relative abundance of sponge symbiotic *Synechococcus* (straight-line graph) and polyP enrichment rate (histogram) in different areas.
