# Supplementary figures and images for: Characteristic Microbiomes Correlate with Polyphosphate Accumulation of Marine Sponges in South China Sea Areas
Source: Microorganisms. 2019 Dec 30;8(1):63. doi: 10.3390/microorganisms8010063 (PMC7022310; doi:10.3390/microorganisms8010063)

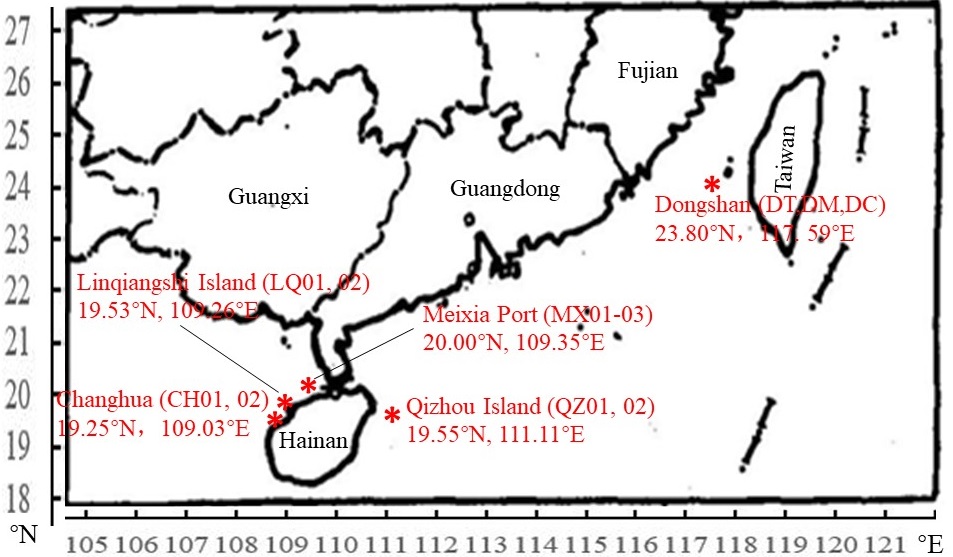

Supplement: Supplementary file 1 [file microorganisms-08-00063-s001.zip › Supplementary Materials/figure S1.jpg]

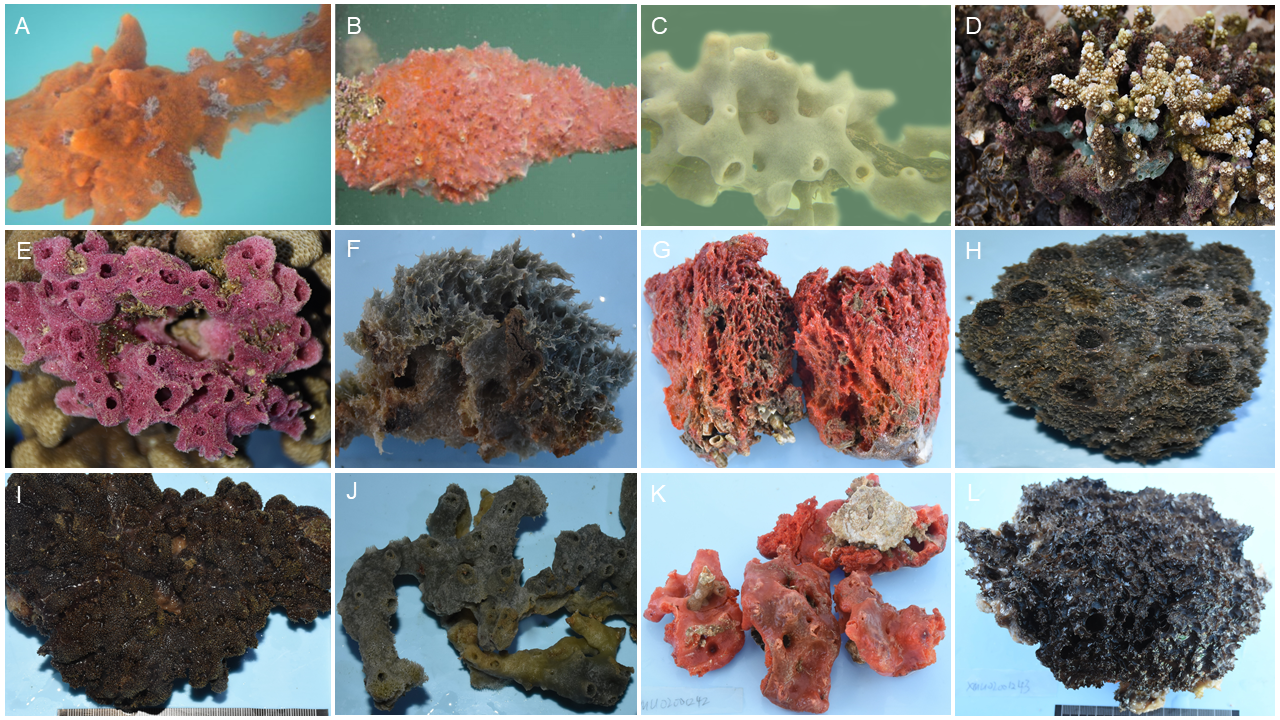

Supplement: Supplementary file 1 [file microorganisms-08-00063-s001.zip › Supplementary Materials/figure S2.jpg]

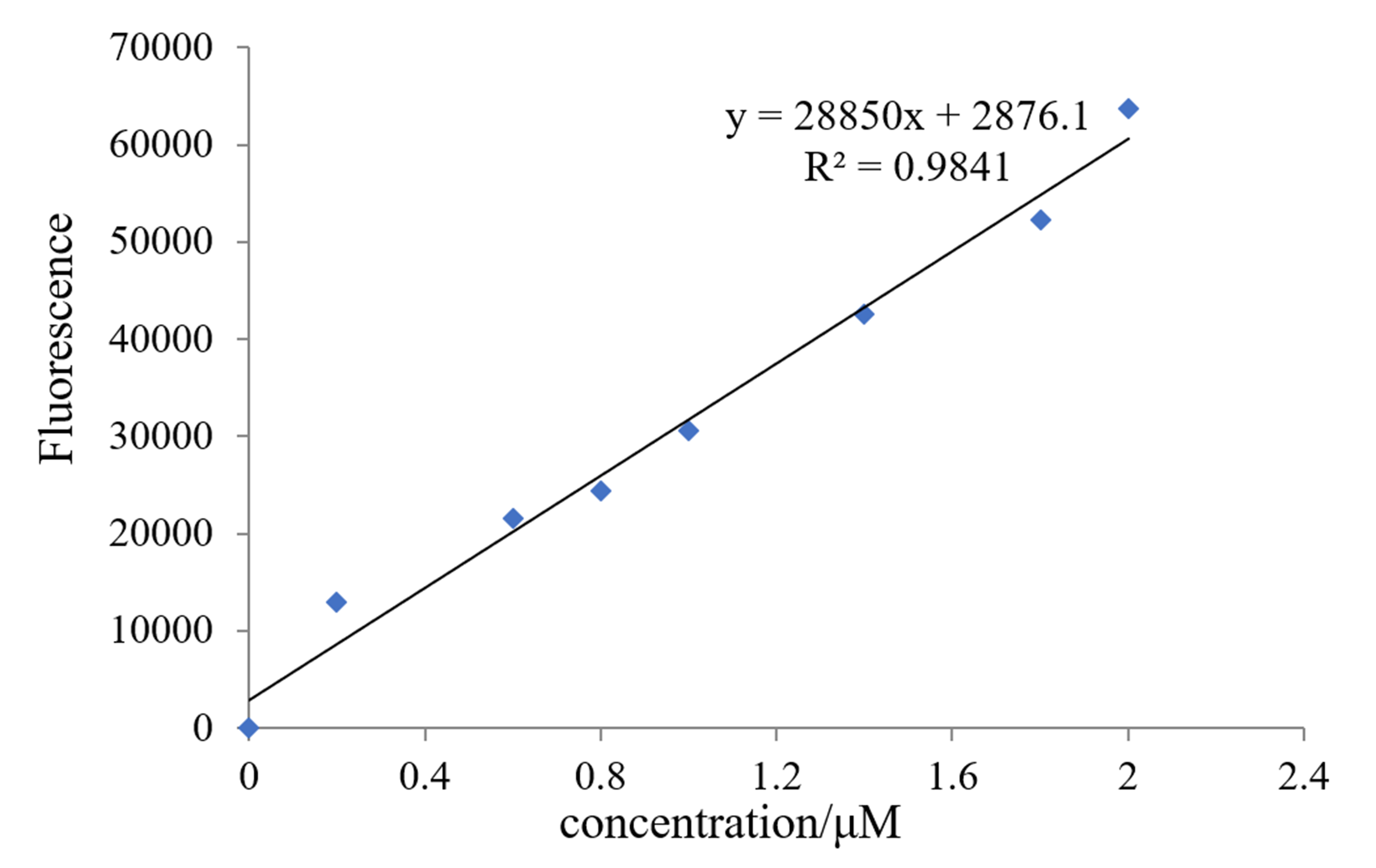

Supplement: Supplementary file 1 [file microorganisms-08-00063-s001.zip › Supplementary Materials/figure S3.jpg]

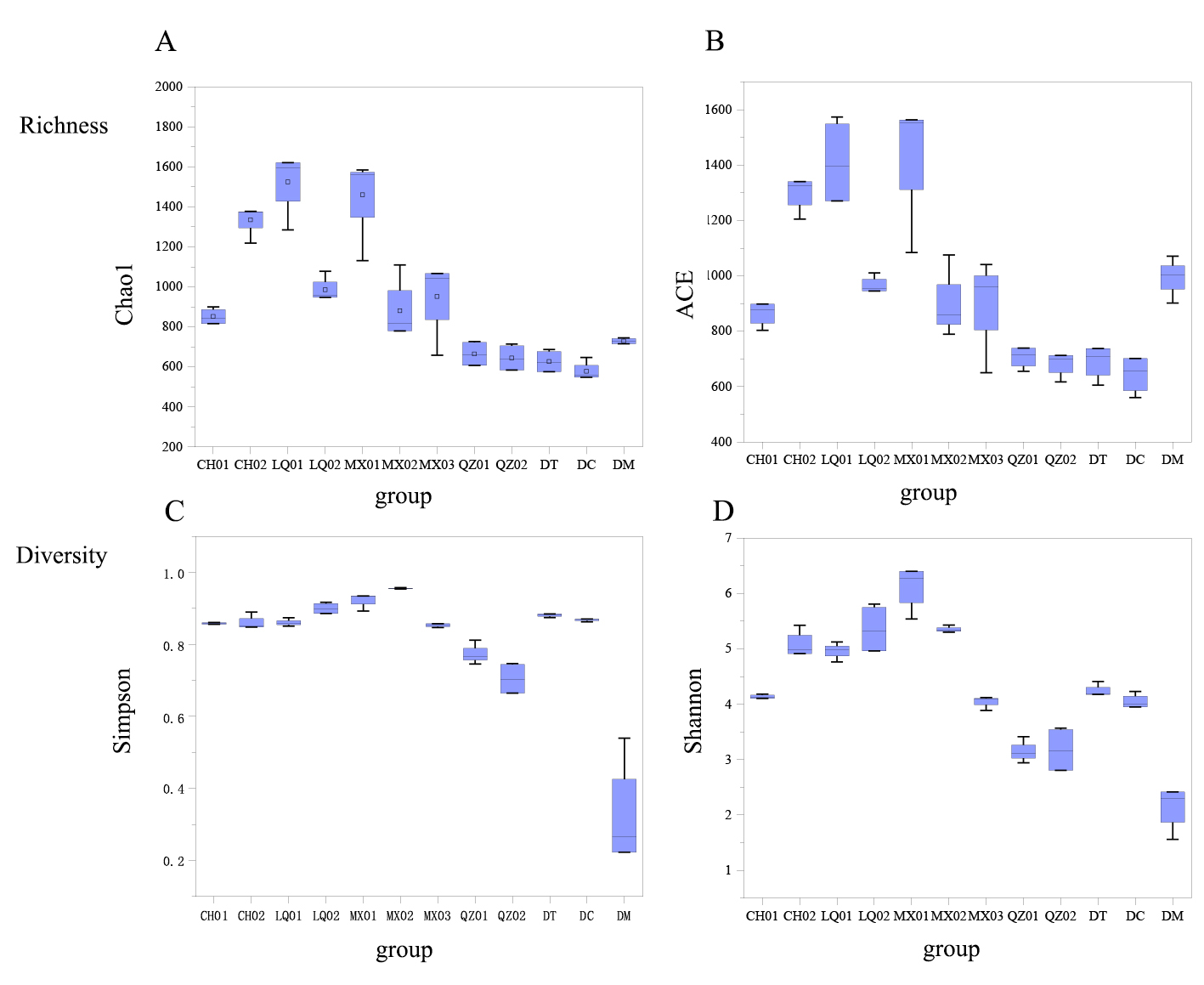

Supplement: Supplementary file 1 [file microorganisms-08-00063-s001.zip › Supplementary Materials/figure S4.jpg]

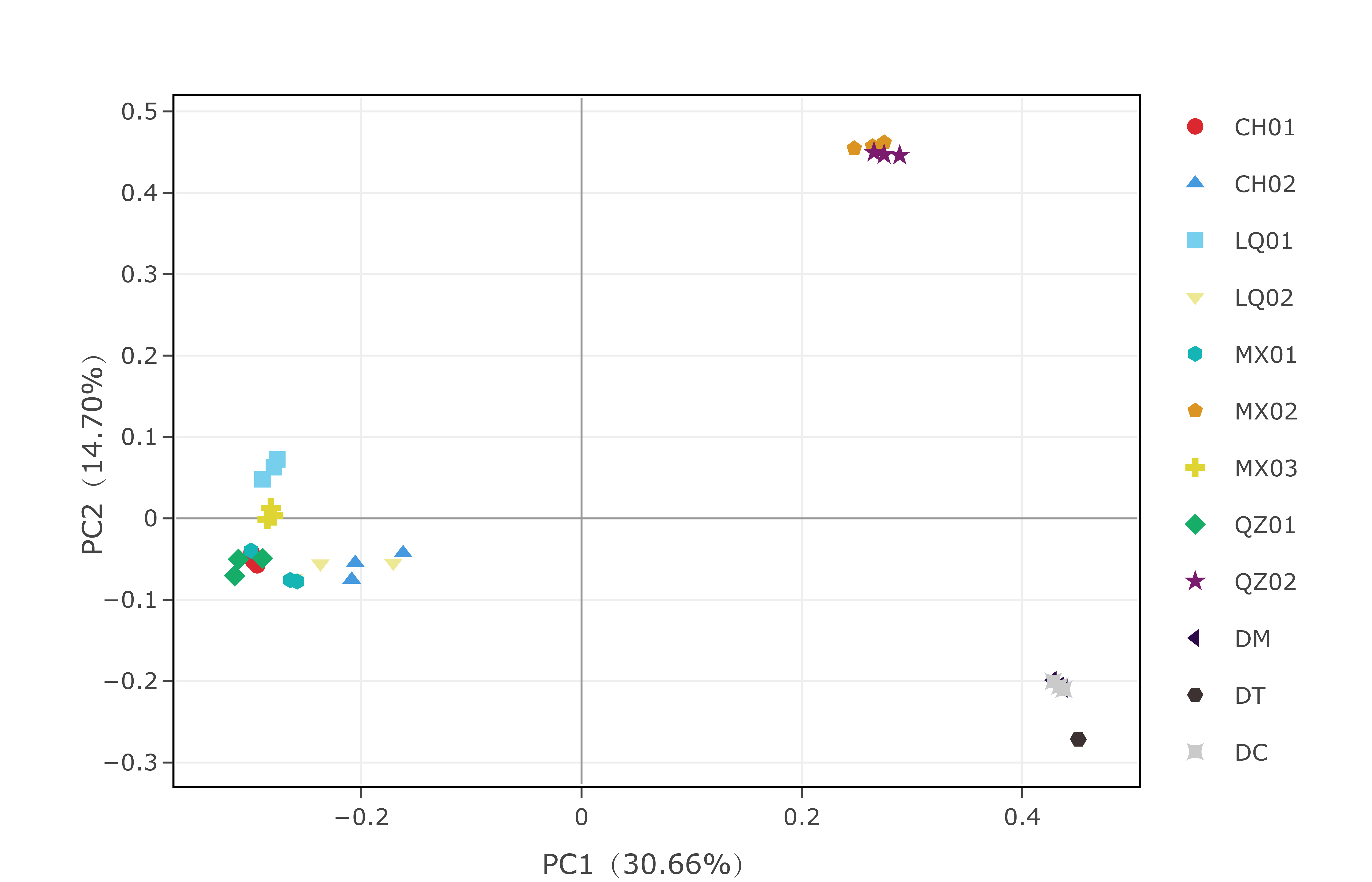

Supplement: Supplementary file 1 [file microorganisms-08-00063-s001.zip › Supplementary Materials/figure S5.jpg]

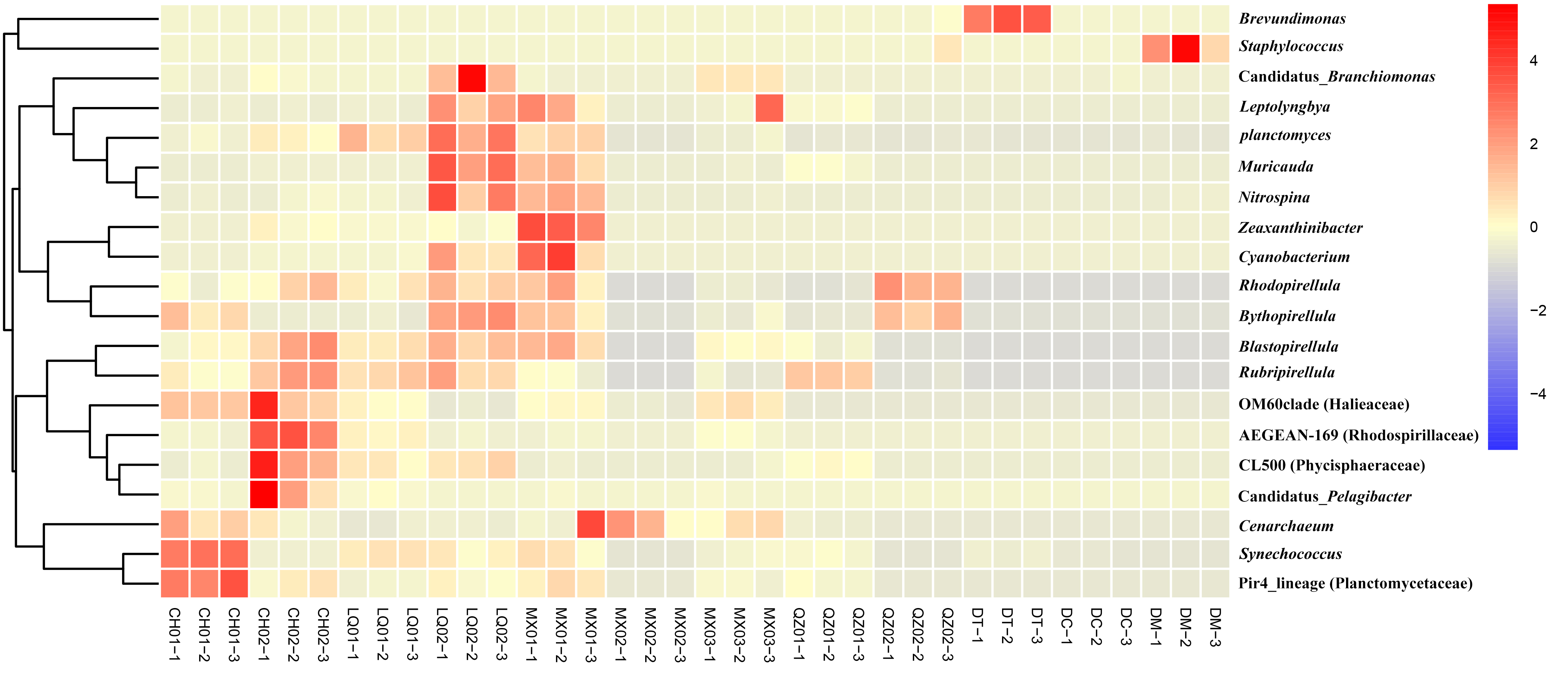

Supplement: Supplementary file 1 [file microorganisms-08-00063-s001.zip › Supplementary Materials/figure S6.jpg]

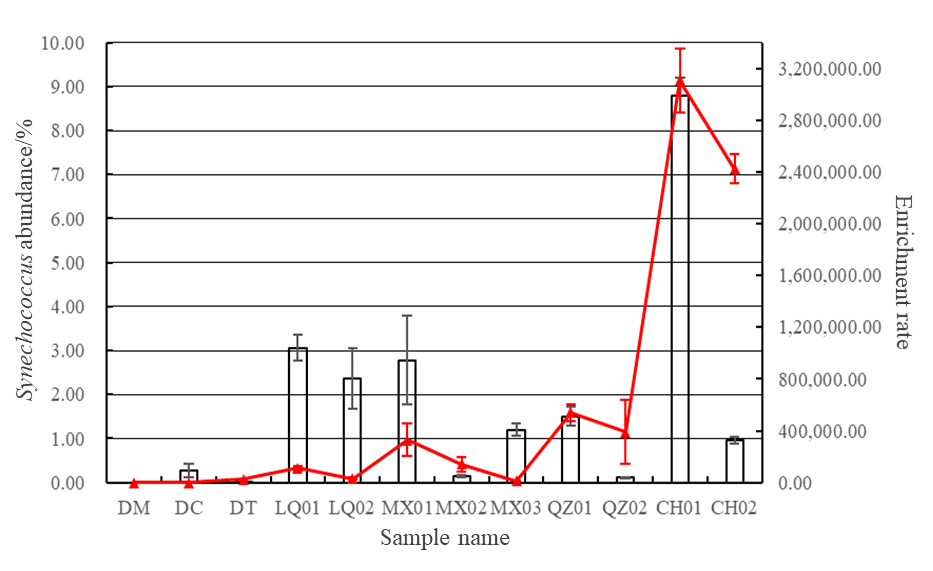

Supplement: Supplementary file 1 [file microorganisms-08-00063-s001.zip › Supplementary Materials/figure S7.jpg]
